# Genome Sequence of Mucoid *Pseudomonas aeruginosa* Strain FRD1

**DOI:** 10.1128/genomeA.00376-15

**Published:** 2015-04-23

**Authors:** Di Wang, Falk Hildebrand, Lumeng Ye, Qing Wei, Luyan Z. Ma

**Affiliations:** a State Key Laboratory of Microbial Resources, Institute of Microbiology, Chinese Academy of Sciences, Beijing, China; b Department of Bioengineering Sciences, Research Group Microbiology, Vrije Universiteit Brussel and VIB Structural Biology Brussels, Brussels, Belgium

## Abstract

*Pseudomonas aeruginosa* is an important opportunistic pathogen. Strain FRD1 is a mucoid isolate from the sputum of a cystic fibrosis patient. It has been widely studied and has many different phenotypes compared to nonmucoid strains. Here, we present the draft genome sequence of *P. aeruginosa* strain FRD1 to gain insight into mucoid isolates.

## GENOME ANNOUNCEMENT

*Pseudomonas aeruginosa* is an important opportunistic pathogen that can cause persistent chronic infections in cystic fibrosis (CF) patients and individuals with a compromised immune system. A mucoid phenotype, a consequence of overproduction of exopolysaccharides alginate, has been found in most *P. aeruginosa* isolates from chronically infected CF patients (1). Strain FRD1, a sputum mucoid isolate from a CF patient, has been well studied over the last few decades (2). Besides being mucoidy, it presents several different phenotypes compared to the nonmucoid *P. aeruginosa* strain, such as loss of motility and lack of lipopolysaccharide (LPS) O-side chains. To better understand the ecological versatility of this important pathogen and the link between genomic complexity and bacterial adaption in human lungs, we sequenced the whole genome of the FRD1 strain and further compared it with nonmucoid strain PAO1 and two other *P. aeruginosa* CF isolates, *Pseudomonas* LESB58 (3) and PA2192 (4).

The FRD1 genome sequence was accomplished using Illumina high throughput reads in a hybrid *de novo* assembly strategy. The open reading frames (ORFs) were predicted using Glimmer 3.02, and gene functions were predicted and classified into different metabolic pathways by BLASTing against the KEGG, COG, and TrEMBL databases. Approximately 368 Mb of raw data were obtained from FRD1 genome sequencing and further assembled into 16 scaffolds containing 31 contigs using SOAPdenovo.

FRD1 has 6.68 Mb of genome that comprises 6,269 genes, which covered 88.44% of the entire genome length. The FRD1 genome is larger than the genome of the nonmucoid PAO1 strain (6.63 Mb) (5), yet its G+C content is 66%, comparable with that of PAO1. Multiple genome alignment by Mauve showed that FRD1 has multiple large inverse fragments compared to that of PAO1. In addition, the FRD1 genome has better identity with CF isolates, strains *Pseudomonas* LESB58 and PA2192, which are both biofilm hyperproducers and devoid of motility (6, 7). Three large predicted gene islands, 1 prephage (~184 kb), and 1 putative cluster of regularly interspaced short palindromic repeats (CRISPR) were found in FRD1. A 67-kb gene island carrying *pvdDIJ* gene clusters was found in both FRD1 and *Pseudomonas* LESB58 strains, but not in PAO1. A 6-kb gene cluster encoding PAGI-7 was only found in FRD1, not in any of the other three strains.

The KEGG pathway showed that the largest group of proteins in FRD1 was involved in environmental processing-membrane transportation. Although FRD1 lacks the LPS B band O-antigen, the FRD1 genome contained all gene clusters for the synthesis of a B band O-antigen, which belongs to the serotype IV subgroup according to BLASTn results (8). FRD1 has genes encoding flagella or type IV pili, two important bacterial surface appendages for swimming and twitching motilities. BLASTp and polygenetic tree analysis (generated by MEGA 4) showed that FRD1 *pilA* (encoded pilin) belongs to group I of 5 *pilA* groups, which has a *pilO* downstream of *pilA* (9).

### Nucleotide sequence accession numbers. 

This whole-genome shotgun project of *Pseudomonas aeruginosa* strain FRD1 has been deposited at GenBank under the accession no. JYJZ00000000. The version described in this paper is the ﬁrst version, JYJZ01000000.
